# Oral Treatment with Extract of *Agaricus blazei* Murill Enhanced Th1 Response through Intestinal Epithelial Cells and Suppressed OVA-Sensitized Allergy in Mice

**DOI:** 10.1155/2011/532180

**Published:** 2010-09-26

**Authors:** Go Bouike, Yosuke Nishitani, Hideyuki Shiomi, Masaru Yoshida, Takeshi Azuma, Takashi Hashimoto, Kazuki Kanazawa, Masashi Mizuno

**Affiliations:** ^1^Department of Agrobioscience, Graduate School of Agricultural Science, Kobe University, 1-1, Rokkodai-cho, Nada-ku, Kobe, 657-8501, Japan; ^2^Health Bioscience Team, Organization of Advanced Science and Technology, Kobe University, Kobe 657-8501, Japan; ^3^Department of Internal Medicine, Graduate school of Medicine, Kobe University, Kobe 650-0017, Japan

## Abstract

To clarify the mechanism of the antiallergic activity of *Agaricus blazei* Murill extract (ABME), the present paper used an *in vivo* allergy model and an *in vitro* intestinal gut model. During OVA sensitization, the serum IgE levels decreased significantly in ABME group. Interleukin (IL)-4 and -5 produced from OVA-restimulated splenocytes was significantly decreased, and anti-CD3*ε*/CD28 antibody treatment also reduced IL-10, -4, and -5 production and increased IFN-*γ* production in ABME group. These results suggest that oral administration of ABME improves Th1/Th2 balance. Moreover, a coculture system constructed of Caco-2 cells and splenocytes from OT-II mice or RAW 264.7 cells indicated that the significant increases in IFN-*γ* production by ABME treatment. Therefore, it was concluded that the antiallergic activity of ABME was due to the activation of macrophages by epithelial cells and the promotion of the differentiation of naïve T cells into Th1 cells in the immune.

## 1. Introduction

There are four types of hypersensitivity disease, and the incidence of type I allergy has been increasing worldwide [1, 2]. Type I allergy is characterized by a high level of immunoglobulin E (IgE) antibodies arising from hypersensitive reactions to allergens such as pollen or food. This incidence caused by environmental factors, exposure to large amounts of antigen, and genetics can affect all age groups at any time in life. In particular, there has been a marked increase in the prevalence of allergies in children and young adults [3].

*Agaricus blazei *Murill is one of the most intensively studied medicinal mushrooms [4–6] among the mushrooms used to treat many diseases [7–11]. It was reported that the extract of *A. blazei *Murill had a potent antitumor activity in mice, and its antitumor activity was postulated to be exerted through mediation of the immune system of the host by *β*-(1, 6)- and *β*-(1, 3)-glucan [5, 6, 12–15]. From these reports, these functions of *A. blazei* Murill have been shown to indirectly affect immune systems.

The mechanism of the pathogenesis of type I allergy is initiated by phagocytosis of allergens by antigen-presenting cells (APC), which represent a part of the antigen on MHC class II molecules to T cell receptors (TCR) on naïve T cells. On the basis of their cytokine production profiles, CD4^+^ T cells can be subdivided into two distinct populations, the T helper type 1 (Th1) and T helper type 2 (Th2) cells [16]. Th2 cells predominantly produce interleukin (IL)-4 and IL-5 [17]. In contrast, Th1 cells mainly secrete cytokines such as IL-2 and interferon (IFN)-*γ* [18, 19]. The balance between Th1- and Th2-dominant immunity (Th1/Th2 balance) is thought to be important for the development of various diseases.

The gut forms a barrier between the internal environment and the outside. Of the various cells that exist in the gut, intestinal epithelial cells (IEC), and macrophages are some of the most important in gut immune systems. The IEC physically prevent the invasion of numerous xenobiotics such as microorganisms and their metabolites from the intestine [20], and, macrophages, which are major APC, play a key role in antigen-specific immunological responses. It has been suggested that the activation of APC is a crucial point in skewing of the balance between Th1 and Th2 immune responses [21]. Intestinal macrophages are major cells in the human mononuclear phagocytic system and are preferentially localized in the subepithelial region [22]. Some studies in gut immune systems have reported that APC are able to select Th1 or Th2 differentiation [23, 24]. In order to investigate the antiallergic effects induced by oral administration of functional foods, it is important to paper the effects of foods that are able to stimulate APC and antigen specific immunological responses through IEC. 

Recently, many reports have shown that *A. blazei* Murill has beneficial effects *in vivo *[12, 25, 26]. However, little information is available on the mechanism of the antiallergic effects induced by oral administration of *A. blazei* Murill. The aim of this study was to clarify the mechanism of the antiallergic effects exhibited after oral administration of* A. blazei* Murill extracts (ABME) using an *in vivo* allergy model mouse and an *in vitro* intestinal gut model.

## 2. Materials and Methods

### 2.1. Reagents and Preparation of *A. blazei* Murill Extract

Dulbecco's Modified Eagle Medium (DMEM), actinomycin D, lipopolysaccharide (LPS) from *E. coli* O127, and murine recombinant tumor necrosis factor (TNF)-*α* were purchased from Wako Pure Chemical Industries (Osaka, Japan). Eagle's Minimum Essential Medium (MEM) was purchased from Nissui pharmaceutical (Tokyo, Japan). RPMI 1640 medium, MEM nonessential amino acids (NEAA), and trypsin were purchased from GIBCO BRL (Grand Island, NY, USA). PiCl was purchased from Tokyo Chemical Industries (Tokyo, Japan). Mouse anti-2,4,6-trinitrophenyl (TNP) monoclonal IgE was purchased from BD pharmingen (San Diego, USA). Ovalbumin (OVA) and Al(OH)_3_ adjuvant were purchased from Sigma (St. Luis, MO, USA). Antimouse CD3*ε* antibody and antimouse CD28 antibody were purchased from Biolegend (San Diego, USA). The other chemicals and reagents were ordinary commercial and guaranteed products. *A. blazei *Murill (Iwade strain 101) extract (ABME) which was composed of hot water extracts from mycelium and fruiting body, and alkaline extracts of fruting body was donated by the Iwade Mushroom Institute (Mie, Japan).

### 2.2. Mice

Female 3-week-old BALB/c mice were purchased from SLC (Shizuoka, Japan). The mice were housed in an air-conditioned animal room at 23 ± 2°C and acclimated for 7 days before the experiments. The mice were fed a laboratory diet (Nihon Nosan, Yokohama, Japan) and water ad libitum. OVA-specific TCR transgenic mice with a C57BL/6J background (OT-II) were purchased from the Jackson Laboratory (Bar Harbor, ME). OT-II mice were originally generated by Barnden et al. [27, 28]. The animal treatments in the present study followed established rules and guidelines approved for animal use and care at Kobe University (The Guidelines for the Care and Use of Experimental Animals of Rokkodai Campus, Kobe University).

### 2.3. IgE-Dependent PCA Reaction in BALB/c Mice

A passive cutaneous anaphylaxis (PCA) reaction was induced with PiCl to determine an approximate drinking concentration of ABME. ABME was suspended in sterilized distilled water at concentrations of 6.0, 1.2, 0.24, and 0.048 mg/ml. Female 5-week-old BALB/c mice were given the extract suspension in their drinking water for 4 days. Then, the mice were passively sensitized by injecting 2 *μ*g/100 *μ*l of mouse anti-TNP monoclonal IgE preparation intravenously into their tail veins. The PCA reaction was evoked by painting 10 *μ*l of 0.8% PiCl acetone-olive oil (1 : 1) solution onto the surface of an earlobe. Ear thickness was measured before and 2 h after the PiCl challenge using a micrometer (Ozaki MFG Co., Ltd, Tokyo, Japan), and edema was calculated according to differences in ear thickness before and after PiCl challenge.

### 2.4. OVA Immunization Protocol in BALB/c Mice

Female 4-week-old BALB/c mice were given the ABME suspension in their drinking water for 32 days. First sensitization was achieved by intraperitoneally injecting 300 *μ*l PBS containing 10 *μ*g OVA mixed with 1 mg Al(OH)_3_  adjuvant. Subsequently, the mice were challenged using an intraperitoneal injection of 300 *μ*l PBS containing 10 *μ*g of OVA mixed with 0.5 mg Al(OH)_3_  adjuvant every 5 days for 4 weeks after the first OVA injection. Some mice were injected with Al(OH)_3 _adjuvant as a control.

### 2.5. Analysis of OVA-Specific and Antigen-Nonspecific Cytokine Responses in BALB/c Mice Splenocytes

Splenocytes were prepared from each mouse followed by Segawa et al. [29]. To determine OVA-specific cytokine production, the hemolyzed splenocytes (5 × 10^6^  cells/ml) were stimulated with 25 *μ*g/ml OVA in a 96-well flat-bottom well microplate for 72 h. After incubation, the culture supernatants were collected for the measurement of cytokines using a Cytometric bead array immunoassay kit (Beckman Coulter, Fullerton, CA, USA) according to manufacturer's protocol.

To determine antigen-nonspecific cytokine production by T cells, 5 *μ*g/ml antimouse CD3*ε* antibody and 5 *μ*g/ml antimouse CD28 antibody were used. The hemolyzed splenocytes in RPMI1640 medium from the OVA-sensitized mice were incubated in a glass dish (diameter: 9 cm, Iwaki) to remove adherent cells. After 2 h incubation, nonadherent cells in the cultured medium were collected and resuspended at a concentration of 5 × 10^6^ cells/ml in RPMI 1640 medium. These nonadherent splenocytes were seeded onto the 96-well microplate (5 × 10^5^ cells/well) containing the antimouse CD3*ε* antibody and 5 *μ*g/ml antimouse CD28 antibody. After incubation for 48 h, the culture supernatants were collected for the measurement of cytokines using the same methods as used for the OVA-specific cytokines.

### 2.6. Blood Samples

Blood samples from each mouse were obtained from the tail vein every 5 days, and whole blood was collected from the postcaval vein on the day of sacrifice. To prepare serum samples, the blood samples were centrifuged at 10,000 rpm for 10 minutes after incubation at room temperature for 30 minutes. Serum samples were stored at −80°C until use.

### 2.7. Cell Culture

Cells of the human intestinal epithelial cell line Caco-2 were cultured in DMEM (high glucose), supplemented with 1% MEM-NEAA, 100 U/ml penicillin, 100 *μ*g/ml streptomycin, and 10% heat-inactivated fetal bovine serum (FBS), and cells of the murine macrophage cell line RAW 264.7 were cultured in DMEM (glutamine, low glucose) supplemented with 10% heat-inactivated FBS, 100 U/ml penicillin, and 100 *μ*g/ml streptomycin. Cells of the murine fibrosarcoma cell line L929 were cultured in MEM supplemented with 10% FBS, 2 mM L-glutamine, 100 U/ml penicillin, and 100 *μ*g/ml streptomycin. All cell cultures were incubated in a humidified 5% CO_2_ incubator at 37°C.

### 2.8. Coculture System Constructed with Caco-2 Cells/OT-II Mice-Derived Splenocytes

To examine the effects of ABME on the OVA-specific response mediated via IEC, Caco-2 cells and splenocytes from OT-II mice were cocultured using Transwell inserts. OT-II mice-derived splenocytes were prepared as described above. Caco-2 cells were seeded at 4.5 × 10^5^ cells/well in RPMI1640 onto transwell insert plates. The cell culture medium was changed every 3 days until the cells were fully differentiated. The splenocytes were suspended at a concentration of 4.5 × 10^5^ cells/well in RPMI1640 (500 *μ*l) in a 24-well tissue culture plate and were incubated for 2 h in a 5% CO_2_ incubator at 37°C to precipitate cells on the plate bottom. The transwell inserts on which the Caco-2 cells had been cultured were then added to the 24-well tissue culture plate. Two hundred microliters of ABME (250 *μ*g/ml) were applied to the apical side for 3 h. After incubation, the inserts were removed, and the splenocytes were restimulated with 10 *μ*g/ml OVA for 72 h. After incubation, the culture supernatants were collected for the measurement of TNF-*α* and IFN-*γ*.

### 2.9. Caco-2/RAW264.7 Cells Coculture System

**Figure 1:**
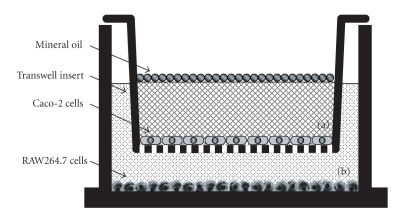
*In vitro* intestinal gut model constructed with Caco-2 cells in apical compartment (a) and RAW264.7 cells in basolateral compartment (b). Transwell inserts on which Caco-2 cells had been cultured were inserted into multiple plate wells containing RAW264.7 cells. Mineral oil (50 *μ*l/well) was added to the surface of the apical compartment (a) to mimic anaerobic conditions.

RAW264.7 cells (4.5 × 10^5^ cells/ml) were plated at 500 *μ*l/well in a 24-well tissue culture plate containing DMEM and cultured for 24 h. The DMEM was removed from the 24-well plate, and transwell inserts containing differentiated Caco-2 cells were placed to the 24-well plates preloaded with RAW264.7 cells. Fresh RPMI 1640 media were used to replace the media in both the apical (a) and basolateral (b) compartments (Figure 1). Furthermore, mineral oil (50 *μ*l/well) was added to the surface of the apical compartment to mimic anaerobic conditions. After 24 h incubation, the mineral oil and culture media of the apical and basolateral compartments were changed for fresh oil and media, and then the cells were incubated. After additional incubation for 12 h, the culture medium in the apical compartment was removed, and RPMI1640 medium containing ABME (250 *μ*g/ml) or LPS (25 *μ*g/ml) was applied for an additional 12 h incubation. The culture supernatants of the basolateral compartment were collected for TNF-*α* and NO measurement.

### 2.10. Cytokines and IgE Content Measurement

TNF-*α* content was quantified using a cytotoxicity assay involving L929 cells (an actinomycin D-treated murine fibroblast cell line) and murine rTNF-*α* as the standard as described by Takada et al. [30]. IFN-*γ*, IL-4, and total IgE contents were measured with ELISA kits (OptEIA set, BD Biosciences, San Diego, CA) in accordance with the manufacturer's instructions. Nitrite as the end product of Nitric oxide (NO) was measured by using Griess reagent (1% sulfanilamide/0.1 % N-1-naphthylethylenediamine dihydrochloride/2.5 % H_3_PO_4_) followed by Green et al. [31].

### 2.11. Immunoprecipitation of ABME with Anti-FIII-2b Polyclonal Antibody

Anti-FIII-2-b polyclonal antibody was prepared as described by Mizuno et al. [32]. The antibody was incubated with ABME (250 *μ*g/ml) or FIII-2-b (250 *μ*g/ml) at 4°C for 2 h, and then the reaction mixture was centrifuged at 7000 rpm for 5 minutes. The supernatant was added to the apical side of the coculture system.

### 2.12. Statistical Analysis

Data are expressed as the mean ± SE. Statistical analysis was performed using the Student's *t*-test. Statistical significance was defined as *P* < .05.

## 3. Results

### 3.1. Suppression of the Anti-TNP IgE-Mediated PCA Reaction by Oral Administration of ABME

**Figure 2:**
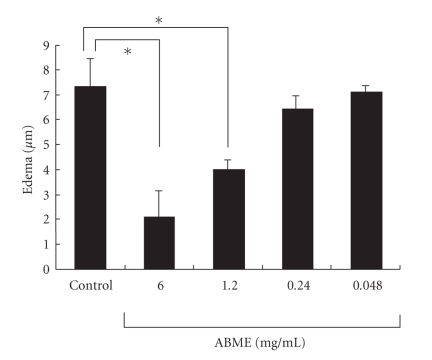
Effects of ABME on the passive cutaneous anaphylaxis reaction in the ears of BALB/c mice. BALB/c mice were given 0.048, 0.24, 1.2, or 6.0 mg/ml ABME in their drinking water, and passive cutaneous anaphylaxis (PCA) was induced as described in Materials and Methods. Edema was measured before and after PiCl challenge. Values represent the means ± S.E. of 4 mice in each group. *P < .05, significantly different from the values of the control group.

The PCA reaction was evoked by painting of PiCl. Oral administration of ABME did not cause any significant difference in weight or drinking amount compared with the control group at any concentration throughout the experiment (data not shown). Furthermore, results demonstrated that the administration of ABME at concentrations of 1.2 and 6.0 mg/ml significantly inhibited edema compared with the control group in a dose-dependent manner (Figure 2).

### 3.2. Downregulation of Serum Immunoglobulin E Levels by Oral Administration of ABME in BALB/c Mice

**Figure 3:**
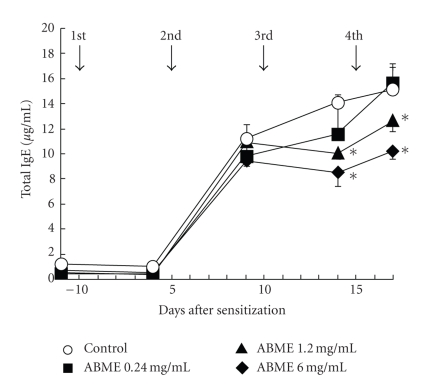
Effect of ABME on OVA sensitization in BALB/c mice The arrows show the days corresponding to the injections of OVA. Serum was obtained from each mouse on the day before each OVA injection, and the level of total IgE was determined by ELISA. Values represent the means ± S.E. of 5 mice in each group. *P < .05, significantly different from the values of the control group.

Antiallergic effects of ABME on allergen-specific responses were examined using BALB/c mice sensitized with OVA. ABME was administered to the mice at the same concentrations in the PCA reaction experiment. Oral administration of ABME did not cause significant changes in weight loss or drinking amount (data not shown). IgE levels increased rapidly after the second injection and then gradually increased until the forth injection in the control group (Figure 3). However, the IgE levels in the ABME-treated group were lower than those in the control group after the third injection, and thereafter they were almost the same. On day 14 of this treatment, the IgE levels in the mice administered 6.0 or 1.2 mg/ml ABME had significantly decreased to 8.5 ± 1.1 and 10.1 ± 0.5 *μ*g/ml, respectively, compared with the control mice (14.1 ± 0.6 *μ*g/ml). These results indicated that oral administration of ABME downregulated the IgE level in serum at concentrations above 1.2 mg/ml.

### 3.3. Inhibitory Effect of ABME on Immune Responses in Splenocytes from OVA-Sensitized BALB/c Mice

**Figure 4:**
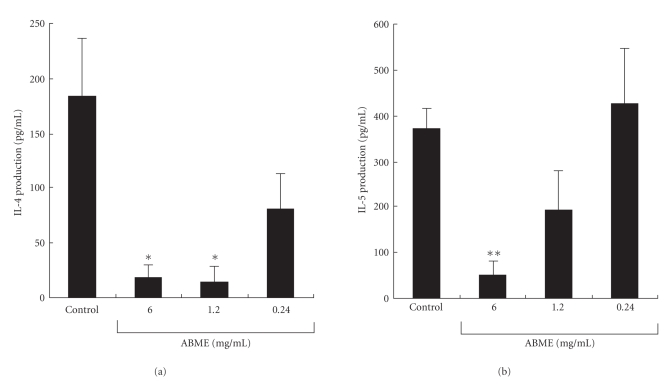
Effect of ABME on the production of the Th2 cytokines IL-4 and 5 from antigen-restimulated splenocytes in OVA-sensitized mice. Two days after the last OVA injection, the spleen cells isolated from each mouse were restimulated with 25 *μ*g/ml OVA. After incubation at 37^o^C for 72 h, the levels of IL-4 (a) and IL-5 (b) in the culture supernatants were determined using a cytometric bead array immunoassay. Values represent the means ± S.E. of 5 mice in each group.*P < .05,**P < .01, significantly different from the values of the control group.

To examine OVA-specific cytokine responses in OVA-sensitized mice, their splenocytes were treated with OVA (25 *μ*g/ml). The IL-4 production in the 6.0 and 1.2 mg/ml ABME groups was significantly decreased to 19.1 ± 11.7 and 15.1 ± 14.0 pg/ml compared with the control group (183.3 ± 53.3 pg/ml) (Figure 4(a)). Furthermore, the IL-5 production was significantly decreased to 50.4 ± 27.5 pg/ml in the 6.0 mg/ml of ABME group compared with the control group (369.1 ± 43.5 pg/ml) (Figure 4(b)). However, IFN-*γ* production, a typical Th1 cytokine, was lower than the detection limit in all groups. These results indicated that oral administration of ABME decreased Th2 response of splenocytes sensitized by OVA. To confirm this, we focused on the T cell antigen receptor signal transduction pathway.

**Figure 5:**
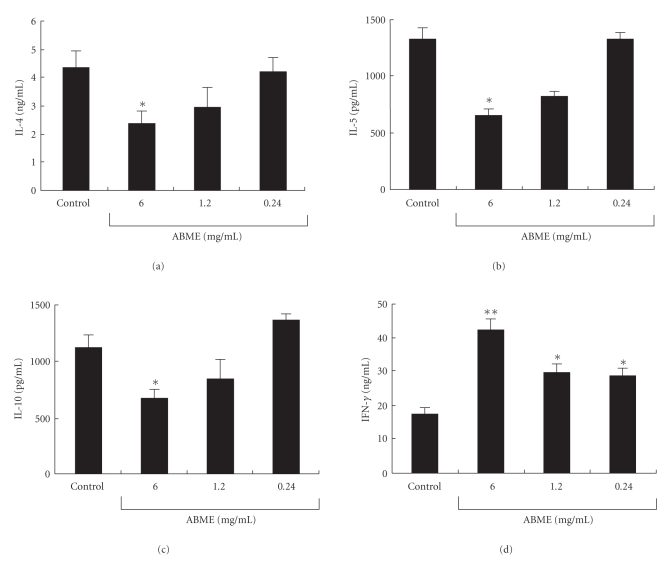
Effect of ABME on the cytokine profile of anti-CD3*ε*/CD28 antibody-stimulated splenocytes in OVA-sensitized mice. Spleen cells from the mice of each group were stimulated with 5 *μ*g/ml each of anti-CD3*ε*/28 antibody at 37°C for 48 h. After incubation, the IL-4 (a), IL-5 (b), IL-10 (c), and IFN-*γ* (d) in the culture medium were determined using a cytometric bead array immunoassay. Values represent the means ± S.E. of 5 mice in each group.*P < .05,**P < .01, significantly different from the values of the control group.

When antigen presenting cells (APC) such as macrophages present an antigenic fragment, T cell activation is dependent on signals delivered through the T cell receptor (TCR)-CD3 complex and the major T cell costimulatory receptor CD28 on the T cell [33–35]. To examine Th1/Th2 cytokine responses mediated via these signaling pathways, spleen cells were treated simultaneously with anti-CD3*ε*- and anti-CD28 antibodies. IL-4, IL-5, and IL-10 production was suppressed by ABME administration in a dose-dependent manner and significantly decreased to 2.4 ± 0.4 ng/ml, 656.2 ± 44.1 pg/ml, and 670.0 ± 70.5 pg/ml, respectively, in 6.0 mg/ml the ABME group (Figures 5(a)–5(c)). On the other hand, IFN-*γ* production was significantly increased compared with the control group (Figure 5(d)). These results suggested that the Th1 response in the splenocytes was increased and the Th2 response was decreased depending on the concentration of ABME ingested when the T cell antigen receptor signal transduction pathway was activated.

### 3.4. Effect of ABME on OVA Stimulated Splenocytes Prepared from OT-II Mice via Caco-2 Cells in CoCulture System

**Figure 6:**
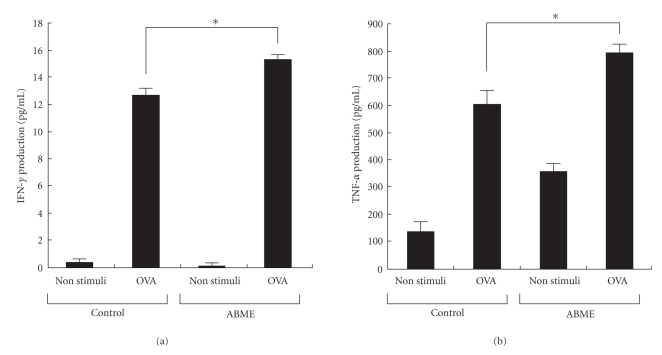
Effect of ABME on INF-*γ* and TNF-*α* production from OVA-stimulated splenocytes prepared from OT-II mice via Caco-2 cells using a coculture system. Splenocytes were cultured in 24-wells plates for 2 h in 5% CO_2_ and incubated at 37°C to precipitate cells on the bottom of the plates. Then, the transwell inserts on which the Caco-2 cells had been cultured were placed into the 24 well plates, which had been preloaded with splenocytes. Two hundred microliters of ABME (250 *μ*g/ml) were applied to the apical side and incubated for 3h. After incubation, the inserts were removed, and the splenocytes were restimulated with 10 *μ*g/ml OVA at 37°C in a CO_2_ incubator for 72 h. After incubation, the culture media were collected for the measurement of IFN-*γ* (a) and TNF-*α* (b) contents as described in Materials and Methods. Values represent the means ± SE. (*n* = 3). *P < .05, significantly different from the values of the control group.

As shown in Figure 5, oral administration of ABME dose-dependently decreased the Th2 response and increased the Th1 response in the spleens of OVA-sensitized mice. In order to investigate how orally administered ABME affects immune cells in the gut, the effect of ABME on IFN-*γ* production was measured in a coculture system constructed of Caco-2 cells and splenocytes prepared from OT-II mice. Since the T cells in splenocytes from OT-II mice highly express an OVA-specific T cell receptor (TCR), the Th1/Th2 cytokine balance can be examined by using OVA as a stimulator. As shown in Figure 6(a), treatment of Caco-2 cells with ABME prior to the stimulation of splenocytes with OVA significantly increased IFN-*γ* production on the basolateral side compared with that observed without ABME stimulation. No significant change was recognized in the coculture system with and without ABME under nonstimulation with OVA. Similarly, ABME pretreatment enhanced TNF-*α* production through Caco-2 cells (Figure 6(b)). These results suggested that the lymphocytes that can produce TNF-*α* were activated by ABME pretreatment via Caco-2 cells, and then, OVA-specific naïve T cells were differentiated into Th1 cells, which can produce INF-*γ*.

### 3.5. Effect of ABME on RAW264.7 Cells via Caco-2 Cells in the Coculture System

**Figure 7:**
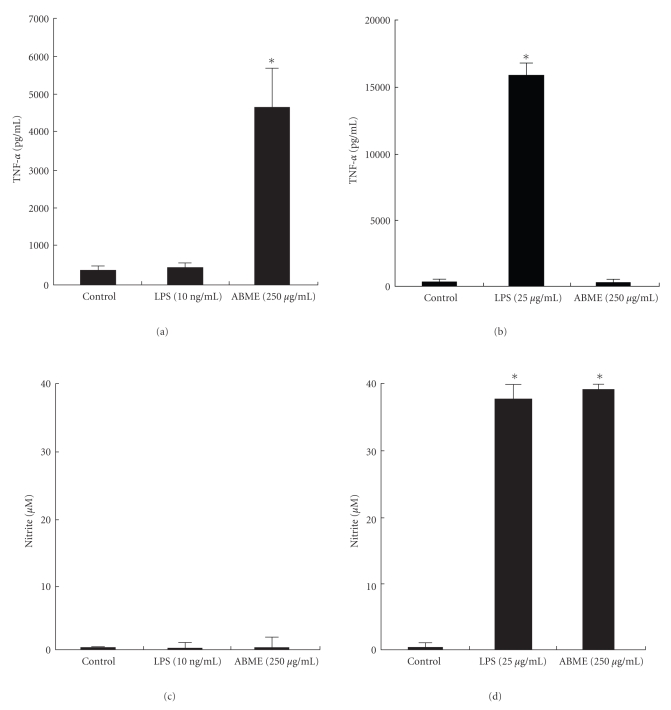
TNF-*α* and NO production in the coculture system or RAW264.7 cells alone treated with ABME or LPS ABME or LPS was added into the apical compartment of the Caco-2/RAW264.7 coculture system (a, c) or added directly into the RAW264.7 cells alone (b, d) and was then incubated for 12 h. After incubation, the supernatants were collected. TNF-*α* and NO secretion into the culture supernatant (basolateral compartment) was determined by a cytotoxicity assay and Griess reagent. Values represent the means ± SE. (*n* = 3). *P < .05, significantly different from the values of the control group.

In the Caco-2/OT-II splenocytes coculture system, ABME induced TNF-*α* production from OVA restimulated splenocytes via Caco-2 cells. TNF-*α* is one of the major cytokines secreted from APC such a macrophages and dendritic cells. Then, the effects of ABME on macrophages were examined using Caco-2/RAW264.7 coculture system. ABME (250 *μ*g/ml) treatment for 12 h to the apical compartment significantly upregulated the TNF-*α* production from RAW264.7 cells (Figure 7(a)). However, ABME did not induced TNF-*α* production from RAW264.7 cells directly (Figure 7(b)). NO production did not induced by ABME treatment (Figure 7(c)), although the direct treatment of ABME to RAW 264.7 did (Figure 7(d)). These results indicate that ABME cannot induce TNF-*α* production from RAW264.7 cells without interacting with Caco-2 cells, and Caco-2 cells can cancel NO production from RAW 264.7 cells.

**Figure 8:**
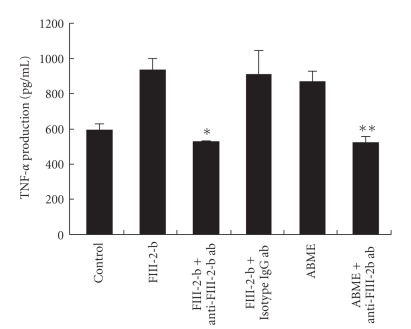
Effect of anti-FIII-2-b antibody on TNF-*α* production induced by ABME in a coculture system. Anti-FIII-2-b antibody or isotype IgG1 (25 ml/well) were incubated with ABME for 2 h and centrifuged at 7000rpm for 5 minutes. The supernatant was added to the apical compartment of the Caco-2/RAW264.7 co-culture system for 3 h. After the incubation, the supernatant from the basolateral side was collected, and TNF-*α* content was measured using a killing assay. Values represent the means ± S.E. (*n* = 3). *P < .05 and **P < .05, significantly different from the values for FIII-2-b and ABME treatment, respectively.

To identify which compounds in ABME enhance TNF-*α* production from RAW264.7 cells, immunoprecipitation using an anti-FIII-2-b antibody was applied. This antibody recognizes the FIII-2-b fraction which was purified as the most active fraction in alkaline-soluble fractions of *A. blazei *Murill [12]. As shown in Figure 8, the anti-FIII-2-b antibody significantly suppressed TNF-*α* production from RAW264.7 to a control levels in a coculture system in which Caco-2 cells were treated FIII-2-b or ABME. This result indicated that the active compound contained in ABME is FIII-2-b.

## 4. Discussion

*A. blazei* Murill is considered to be one of the most important edible and medicinal mushrooms in Japan. It was traditionally used for the treatment of many common diseases like atherosclerosis, hepatitis, hyperlipidemia, diabetes, dermatitis, cancer, and allergy disease [15]. Choi et al. [26] demonstrated that the water extract of the *A. blazei* Murill fruiting body suppressed allergic edema after oral administration and reduced histamine release by direct incubation with mast cells. In general, recent studies are focusing on elucidation action mechanisms for mushrooms extracts, including ABME, hence this study on the mechanism for the antiallergic effects exhibited after oral administration of ABME *in vivo* and *in vitro* systems.

The PCA reaction is a simple method for assessing the inhibitory effects of orally administered compounds on type I allergy. Many beneficial foods and components such as tea leave saponins [36] and mushrooms [10, 37] have been demonstrated antiallergic effects using this method. In this study, ABME suppressed PiCl-induced edema in a dose-dependent manner (Figure 2). Moreover, ABME exerted a significant suppressive effect on the serum IgE levels of OVA-sensitized mice at concentrations of 1.2 mg/ml and above (Figure 3). Similarly, Segawa et al. [29] demonstrated that oral administration of heat-killed *Lactobacillus brevis* inhibited total IgE production by improving the Th1/Th2 balance by enhancing IL-12 and IFN-*γ* production and inhibiting IL-4 production from OVA-sensitized mice splenocytes. It was demonstrated that the levels of IL-4 and IL-5 secreted from splenocytes restimulated with OVA in the 6.0 and 1.2 mg/ml ABME groups were lower than those in the control groups (Figure 4). Furthermore, restimulation with anti-CD3*ε*/CD28 antibodies diminished IL-10 secretion but increased IFN-*γ* production in addition to decreases of IL-4 and IL-5 production (Figure 5). Consequently, these results suggested that the oral administration of ABME shifted the Th1/Th2 balance towards Th1 and downregulated IgE levels. It was reported that ABME enhanced IL-12 and IL-18 mRNA expression in macrophages [38]. Therefore, ABME may affect APC to modulate the Th1/Th2 balance.

It has been found that the gut immune cells are shifted to the Th2 type in allergic patients [24]. As various immune cells are affected by IEC in the gut [39], we used a coculture system using splenocytes from OT-II mice, which possess CD4^+^ T cells that highly express the OVA-specific TCR, and Caco-2 cells for assessing the effects of ABME on the differentiation of T cells via IEC. IFN-*γ* and TNF-*α* production was significantly increased in the ABME- and OVA-stimulated coculture system as compared to that without ABME treatment (Figure 6). IFN-*γ* is secreted from Th1 cells and promotes the proliferation of Th1 cells [40]. TNF-*α* is one of the major cytokines secreted from activated APC [41–43]. Considering these results, our hypothesis is that ABME activates APC by interacting with IEC and then induces Th1 differentiation of naïve T cells in splenocytes by increasing the expression of Th1 cytokines and decreasing that of Th2 cytokines. This is the first report to show the effects of ABME via IEC. To confirm this hypothesis in which ABME activates APC through IEC, we examined whether ABME could activate macrophages in a slightly modified coculture system constructed of Caco-2 and RAW264.7 cells [44]. ABME enhanced TNF-*α* production from RAW264.7 cells in the coculture system but did not induce it during direct treatment of RAW264.7 cells alone. Moreover, nitric oxide was not detected in coculture system, albeit direct treatment of ABME to RAW 264.7 produced it (Figure 7). Recently, Nakao et al. [45] reported that hydrogen peroxide (H_2_O_2_) induced the production of TNF-*α* in RAW 264.7 cells and did not support nitric oxide production unlike LPS. These results may suggest that ABME may indirectly affect immune cells such as macrophages through IEC which is produces H_2_O_2_ as a second messenger. However, further investigations are necessary to clarify how ABME activates macrophages through IEC.

It was demonstrated that ABME suppressed IgE content in OVA-sensitized mice by shifting the Th1/Th2 balance to Th1 and that IEC are absolutely imperative for activating APC, which induce the differentiation of naïve T cells to Th1 type cells. It has been reported that *A. blazei* Murill contain *β*-glucans including the FIII-2-b fraction [6, 12–14]. We showed that TNF-*α* production from the basolateral side of the coculture system was decreased to control levels when this antibody was pretreated with FIII-2-b or ABME solution (Figure 8). This result suggested that the active compound in ABME producing its antiallergic effect is FIII-2-b. 

**Figure 9:**
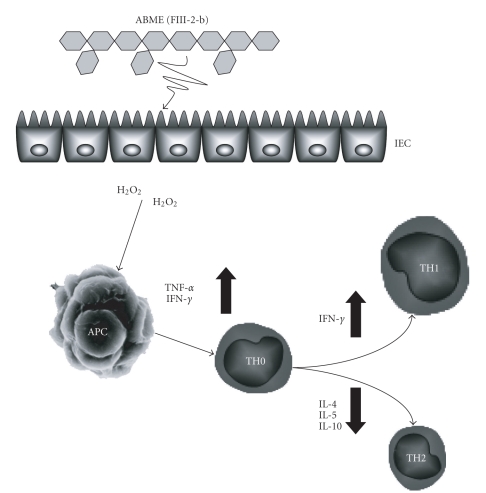
Shift in Th1/Th2 balance to Th1 by ABME through intestinal epithelial cells. An intestinal epithelial cell is stimulated by FIII-2-b in ABME, which binds to a certain receptor. This stimulus produces second messengers, likely H_2_O_2_. Macrophages result in enhancement of TNF-*α* and IFN-*γ* production. Produced INF-*γ* affects Th1/Th2 balance which is important for allergy, and promotes the negative regulation of IL-4, 5, 10 which induce Th2 cell dominance, resulting in Th1 cell dominance in immune system.

In conclusion, this paper demonstrates that FIII-2-b in ABME activates macrophages through IEC, but without them. These findings suggest that an activation process linked to macrophages is involved IEC. H_2_O_2_ which was produced from Caco-2 plays an important role of a second messenger to produce TNF-*α* from macrophages and is involved in the signal transduction to promote the differentiation of naïve T cells into Th1 cells (Figure 9).
